# Anti-inflammatory and anti-oxidant mechanisms of an MMP-8 inhibitor in lipoteichoic acid-stimulated rat primary astrocytes: involvement of NF-κB, Nrf2, and PPAR-γ signaling pathways

**DOI:** 10.1186/s12974-018-1363-6

**Published:** 2018-11-23

**Authors:** Eun-Jung Lee, Jin-Sun Park, Yu-Young Lee, Do-Yeon Kim, Jihee Lee Kang, Hee-Sun Kim

**Affiliations:** 1 Department of Molecular Medicine, Tissue Injury Defense Research Center, School of Medicine, Ewha Womans University, Mok-6-dong 911-1, Yangchun-Ku, Seoul, 158-710 South Korea; 2 Department of Physiology, Tissue Injury Defense Research Center, School of Medicine, Ewha Womans University, Seoul, South Korea

**Keywords:** MMP-8 inhibitor, Astrocytes, Neuroinflammation, Anti-inflammatory, Antioxidant, Molecular mechanisms

## Abstract

**Background:**

Recent evidence suggests that reactive astrocytes play an important role in neuroinflammation and neurodegenerative diseases. Thus, controlling astrocyte reactivity has been suggested as a promising strategy for treating neurodegenerative diseases. In the present study, we investigated whether a matrix metalloproteinase (MMP)-8 inhibitor, M8I, could control neuroinflammation in lipoteichoic acid (LTA)-stimulated rat primary astrocytes.

**Methods:**

The effects of M8I on the expression of inducible nitric oxide synthase, cytokines, and MMPs were examined in LTA-stimulated rat primary astrocytes by ELISA, RT-PCR, and Western blot analysis. The effects of M8I on reactive oxygen species (ROS) generation and phase II antioxidant enzyme expression were examined by the DCF-DA assay, RT-PCR, and Western blot analysis. The detailed molecular mechanisms underlying the anti-inflammatory and antioxidant effects of M8I were analyzed by the electrophoretic mobility shift assay, the reporter gene assay, Western blot, and RT-PCR analysis.

**Results:**

Treatment with LTA, a major cell wall component of Gram-positive bacteria, led to astrocyte activation and induced the expression of inflammatory molecules such as iNOS, COX-2, and pro-inflammatory cytokines. In addition, LTA induced the expression of MMPs such as MMP-1, MMP-3, MMP-8, MMP-9, and MMP-13 in rat primary astrocytes. Based on previous reports showing that MMP-8 plays a role as a proinflammatory mediator in microglia, we investigated whether MMP-8 is also involved in inflammatory reactions of reactive astrocytes. We found that treatment of astrocytes with M8I significantly inhibited LTA-induced expression of iNOS, TNF-α, IL-1β, IL-6, and TLR-2. In addition, M8I inhibited LTA-induced NF-κB, MAP kinase, and Akt activities, while it increased the anti-inflammatory PPAR-γ activities. Moreover, M8I showed antioxidant effects by suppressing ROS production in LTA- or H_2_O_2_-stimulated astrocytes. Interestingly, M8I increased the expression of phase II antioxidant enzymes such as hemeoxygenase-1, NQO1, catalase, and MnSOD by modulating the Nrf2/ARE signaling pathway.

**Conclusions:**

The data collectively suggest the therapeutic potential of an MMP-8 inhibitor in neuroinflammatory disorders that are associated with astrocyte reactivity.

**Electronic supplementary material:**

The online version of this article (10.1186/s12974-018-1363-6) contains supplementary material, which is available to authorized users.

## Background

Astrocytes are the most abundant cell type in the central nervous system (CNS) and play crucial roles in brain development, synaptic transmission, regulation of blood flow, ion homeostasis, and energy metabolism [1, 2]. Insults to the CNS trigger the release of numerous factors that interact with astrocytes and trigger reactive gliosis, which is generally represented by hypertrophy and astrocyte proliferation. Recent studies have shown that there are two distinct types of reactive astrocytes, termed A1 and A2, which may be harmful or beneficial, respectively [3]. A1 astrocytes are rapidly induced by CNS injury and release neurotoxic factors such as glutamate, reactive oxygen species (ROS), and cytokines [4, 5]. In contrast, A2 astrocytes are induced by ischemia and express neurotrophic factors, promoting repair processes that protect against the effects of brain injury [3]. The reactive astrocytes seem to exist as a continuous spectrum of progressive changes with a mixed population. Recent studies reported that reactive astrocytes are found in vulnerable brain regions of animal models and patients with neurodegenerative diseases including Alzheimer’s disease (AD), Parkinson’s disease (PD), Huntington’s disease (HD), amyotrophic lateral sclerosis (ALS), and multiple sclerosis (MS), suggesting the role of astrocytes in the pathogenesis of these diseases [6, 7].

Previous studies have reported that Gram-positive bacterial infections of the CNS occur in bacterial meningitis and trigger brain inflammatory responses [8, 9]. In the CNS, astrocytes and microglia are considered targets of Gram-positive bacterial infection [10]. Lipoteichoic acid (LTA) is a major component of the Gram-positive bacterial cell wall that induces glial inflammatory activation, which is mediated through toll-like receptor 2 (TLR2) signaling [9, 11]. TLR2 is also involved in glial cell activation detected in non-infectious neurological disorders [12]. Several studies have demonstrated that TLR2 functions as a sentinel receptor to detect neuronal cell death and tissue damage in neurological conditions such as traumatic brain injury, intracerebral hemorrhage, and hippocampal excitotoxicity [13–15]. In addition, TLR2 on microglia functions as a receptor for fibrillary Aβ peptide and oligomeric α-synuclein and thereby modulates neuroinflammation during AD and PD [16, 17]. Thus, TLR2 has been suggested as an efficient target to regulate unwanted inflammatory responses in neurological disorders.

Matrix metalloproteinases (MMPs) belong to zinc-containing endopeptidases that regulate cell-matrix composition and processing of bioactive molecules [18]. Under pathological conditions, aberrant expression of MMPs induces chronic inflammation and contributes to the progression of neurodegenerative diseases [19]. Several studies have demonstrated that MMP-8, also known as neutrophil collagenase, is involved in neuroinflammatory disorders such as bacterial meningitis, spinal cord injury, and multiple sclerosis [20–22]. Our group recently reported that MMP-8 plays a pivotal role in neuroinflammation via tumor necrosis factor (TNF)-α modulation in microglia [23]. We also demonstrated that MMP-8 is a novel pathogenic factor in focal cerebral ischemia [24]. Furthermore, MMP-8 mediates neuroinflammation in aged normal and LRRK2 G2019S Parkinson’s disease model mice challenged with lipopolysaccharide (LPS) [25].

Although previous studies have demonstrated the role of MMP-8 in microglia [23–25], the role of MMP-8 in reactive astrocytes has not been clearly demonstrated. Therefore, in the present study, we examined the effect of the MMP-8 inhibitor in LTA-stimulated primary astrocytes and analyzed detailed molecular mechanisms.

## Methods

### Reagents and antibodies

All cell culture reagents and antibiotics were purchased from Gibco BRL (Grand Island, NY, USA). Purified lipoteichoic acid (LTA) from *Staphylococcus aureus* was obtained from InvivoGen (San Diego, CA, USA). The MMP-8 inhibitor, M8I, was purchased from Calbiochem (La Jolla, CA, USA). All reagents and enzymes for reverse transcription polymerase chain reaction (RT-PCR) and oligonucleotides for the electrophoretic mobility shift assay (EMSA) were purchased from Promega (Madison, WI, USA). Antibodies against phospho-/total forms of MAP kinases, β-actin, and MMP-8 were supplied by Cell Signaling Technology (Danvers, MA, USA) and Abcam (Cambridge, UK). Antibodies against HO-1, NQO1, catalase, MnSOD, Nrf2, c-Jun, and lamin A were purchased from Santa Cruz Biotechnology (Santa Cruz, CA, USA). The antibody for phospho-p47^phox^ (Ser370) was purchased from Assay Biotechnology Company Inc. (Sunnyvale, CA, USA). All other chemicals were obtained from Sigma-Aldrich, unless otherwise stated.

### Rat primary astrocyte cultures and cell viability test

Primary astrocytes were cultured from the cerebral cortices of postnatal day 1 Sprague-Dawley rat pups as described previously [26]. In brief, after cortices were dissected from rat pups, cells were dissociated by pipetting through pores of different sizes and resuspended in minimum essential medium containing 10% fetal bovine serum, penicillin (10 U/ml), streptomycin (10 μg/ml), 2 mM glutamine, and 10 mM 4-(2-hydroxyethyl)-1-piperazineethanesulfonic acid (HEPES). Cell suspensions were plated in poly-D-lysine (1 μg/ml)-coated T75 flasks and incubated at 37 °C under 5% CO_2_. Seven days later, the culture flasks were shaken at 280 rpm for 16 h to remove microglia and oligodendrocytes. The remaining astrocytes were trypsinized and seeded onto a culture plate and incubated for 6 days, with media being changed every 2 days. The purity of astrocyte cultures was > 95%, as confirmed by Western blot and immunocytochemistry analyses using an antibody against glial fibrillary acidic protein. Cell viability was determined by 3-(4,5-dimethylthiazol-2-yl)-2,5-diphenyl tetrazolium bromide (MTT) reduction assay, as previously described [27].

### Measurement of cytokines and nitrite levels

Primary astrocytes (2 × 10^5^ cells per well in a 24-well plate) were pre-treated with M8I for 1 h and further stimulated with LTA (10 μg/ml) for 24 h. The levels of TNF-α and IL-6 released into the media were determined using ELISA kits according to the manufacturer’s instructions (BD Biosciences, San Jose, CA, USA). Nitrite levels in the conditioned media were measured using Griess reagent (Promega). Absorbance at 550 nm was measured with a microplate reader (Molecular Devices, Sunnyvale, CA, USA).

### Determination of ROS generation

A 2′,7′-dichlorofluorescein diacetate (DCF-DA) assay was performed to observe the intracellular ROS level. Cells were plated in a 24-well plate and exposed to assigned doses of M8I and then stimulated with LTA (10 μg/ml) or H_2_O_2_ (500 μM). After 30 min, the cells were stained with 50 μM H_2_DCFDA and incubated at 37 °C for 30 min in the dark. The fluorescent intensity of cells was detected at 485 nm excitation and 535 nm emission by fluorescence plate reader (Molecular Devices).

### Assay for MMP-8 activity

Primary astrocytes (2 × 10^5^ cells per well in a 24-well plate) were treated with M8I prior to LTA stimulation for 1 h, and the supernatants were collected to measure MMP-8 activity. The total MMP-8 activity was measured using the SensoLyte® 520 MMP assay system (AnaSpec, San Jose, CA, USA) as described previously [28].

### Reverse-transcription polymerase chain reaction

Total RNA (1 μg) isolated from primary astrocytes was reverse transcribed, and synthesized cDNA was used as a template for PCR. Reverse-transcription polymerase chain reaction (RT-PCR) was performed on a T100 Thermal cycler (Biorad Laboratories, Hercules, CA, USA) with Go Taq polymerase (Promega). The gene-specific primers used are listed in Table 1. PCR products were visualized by ultraviolet illumination after electrophoresis. The amplified products were normalized using GAPDH as an internal control, and their values were calculated as fold change relative to control.

**Table 1:** Primers used in RT-PCR reactions

Gene	Forward primer (5′ → 3′)	Reverse primer (5′ → 3′)	Size
TNF-α	AAGTTCCCAAATGGGCTCCCT	TGAAGTGGCAAATCGGCTGAC	306 bp
iNOS	GCAGAATGTGACCATCATGG	ACAACCTTGGTGTTGAAGGC	426 bp
IL-1β	AAATGCCTCGTGCTGTCTGACC	TCCCGACCATTGCTGTTTCCT	377 bp
IL-6	GAGTTCCGTTTCTACCTGGA	AGCCACTCCTTCTGTGACTC	200 bp
COX-2	TCAGGAAGT TCCTTATTTCCTTTC	TGCGATGCTCTTCCGAGCTGTGCT	479 bp
TLR2	ATGAGGTTCTCCACCCAATA	AGACTCTGGAAGCAGGTGAC	400 bp
MMP-1	GCCATTACCAGTCTCCGAGGA	GGAATTTGTTGG CATGACTCTCAC	467 bp
MMP-2	GATCTGCAAGCAAGACATTG	AAGTGCTGGCAGAATAGACC	242 bp
MMP-3	GTACCAACCTATTCCTGGTTGC	CCAGAGAGTTAGATTTGGTGGG	231 bp
MMP-8	TACAACCTGTTTCTCGTGGCTGC	TCAACTGTTCTCAGCTGGGGATG	317 bp
MMP-9	AAGTTGAACTCAGCCTTTGAGG	GTCGAATTTCCAGATACGTTCC	225 bp
MMP-13	CGTAGTGATCAGAGCCAAGC	TCTGCCTTTCCTGCAATTAGA	225 bp
PPARγ	CCGAAGAACCATCCGATT	CGGGAAGGACTTTATGTA	271 bp
HO-1	ATACCCGCTACCTGGGTGAC	TGTCACCCTGTGCTTGACCT	209 bp
NQO1	ATCACCAGGTCTGCAGCTTC	GCCATGAAGGAGGCTGCTGT	210 bp
Catalase	CCTGACATGGTCTGGGACTT	CAAGTTTTTGATGCCCTGGT	201 bp
MnSOD	GGCCAAGGGAGATGTTACAA	GAACCTTGGACTCCCACAGA	216 bp
Nrf2	AGCAGGACATGGATTTGATT	CTTCTCCTGTTCCTTCTGGA	164 bp
c-Jun	AAGAACTCGGACCTTCTCAC	CTGGCTATGCAGTTCAGCTA	207 bp
GAPDH	GTGCTGAGTATGTCGTGGAGTCT	ACAGTCTTCTGAGTGGCAGTGA	292 bp

### Western blot analysis

Equal amounts of protein were separated by SDS-PAGE and transferred to nitrocellulose membranes, and the membranes were blocked using 5% skim milk in Tris-buffered saline with Tween-20 (TBST). After 1 h, the membranes were incubated with primary antibodies against MMP-8 (1:1000, Abcam), the phospho- or total form of MAP kinases (1:1000, Cell Signaling), HO-1, NQO1, MnSOD, catalase, Nrf2, c-Jun, and lamin A (1:1000, Santa Cruz) at 4 °C overnight. Membranes were incubated with horseradish peroxidase-conjugated secondary antibodies (1:2000 dilution in TBST; Bio-Rad Laboratories), and an enhanced chemiluminescence detection kit was used for evaluations (Thermo Fisher Scientific, Waltham, MA).

### Transient transfection and luciferase assay

Primary astrocytes (2 × 10^5^ cells per well in a 12-well plate) were transfected with 1 μg of plasmid DNA ([κB]_3_-luc, ARE-luc, PPRE-luc) using Metafectene®Pro transfection reagent (Biontex, Munich, Germany). After 36 h of transfection, cells were pretreated with M8I for 1 h and treated with LTA (10 μg/ml) for 6–16 h. Then, cells were harvested and the luciferase assay was performed as previously described [26].

### Electrophoretic mobility shift assay

Nuclear extracts from astrocytes were prepared as previously described [28]. Double-stranded DNA oligonucleotides containing the NF-κB or ARE consensus sequences (Promega) were end-labeled using T4 polynucleotide kinase (New England Biolabs, Beverly, MA) in the presence of [γ-^32^P]ATP. Nuclear proteins (5 μg) were incubated with a ^32^P-labeled probe on ice for 30 min and resolved on a 5% acrylamide gel.

### Statistical analysis

Unless otherwise stated, all experiments were performed with triplicate samples and repeated at least three times. Data are presented as mean ± standard error of the mean (S.E.M.), and statistical comparisons among groups were performed using one-way ANOVA followed by Newman-Keuls post hoc tests or *t* tests. Statistical significance was accepted for *p* values < 0.05.

## Results

### LTA induces the expression of proinflammatory cytokines, iNOS, COX-2, TLR2, and MMPs in rat primary astrocytes

**Fig. 1:**
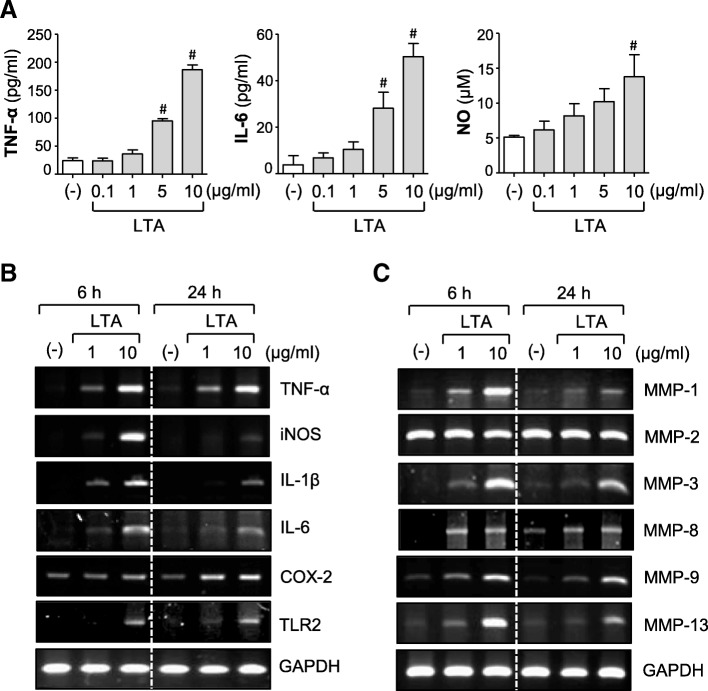
LTA treatment increased the expressions of iNOS, COX-2, cytokines, TLR2, and MMPs in astrocytes. **a** Cells were treated with LTA (0.1, 1, 5, or 10 μg/ml) for 24 h. The amounts of TNF-α, IL-6, and NO were measured in the supernatants. The data are the mean ± S.E.M. of three independent experiments. ^#^*P* < 0.05, vs. control samples. **b**, **c** Cells were treated with LTA (1 or 10 μg/ml) for 6 h or 24 h. RT-PCR was performed to measure the mRNA expression of **b** iNOS, cytokines, COX-2, TLR-2, and **c** six types of MMPs. The data are representative of three independent experiments

To determine whether astrocytes are activated after LTA exposure, rat primary cultured astrocytes were treated with LTA (0.1–10 μg/ml) for 6–24 h. We found that LTA significantly increased the production of NO and proinflammatory cytokines such as TNF-α and IL-6 in a concentration-dependent manner (Fig. 1a). In addition, LTA increased the mRNA expression of proinflammatory mediators, such as TNF-α, IL-1β, IL-6, iNOS, and COX-2 (Fig. 1b and Additional file 1: Figure S1). Interestingly, we found that LTA increased TLR2 mRNA expression, suggesting that LTA contributes to the regulation of its recognition receptor, TLR2. LTA also upregulated the mRNA expression of MMP-1, MMP-3, MMP-8, MMP-9, and MMP-13 without affecting MMP-2 (Fig. 1c and Additional file 1: Figure S1). These data indicate that LTA induces the expression of proinflammatory mediators by activating astrocytes.

### Inhibition of MMP-8 suppresses the production of proinflammatory molecules in LTA-stimulated astrocytes

**Fig. 2:**
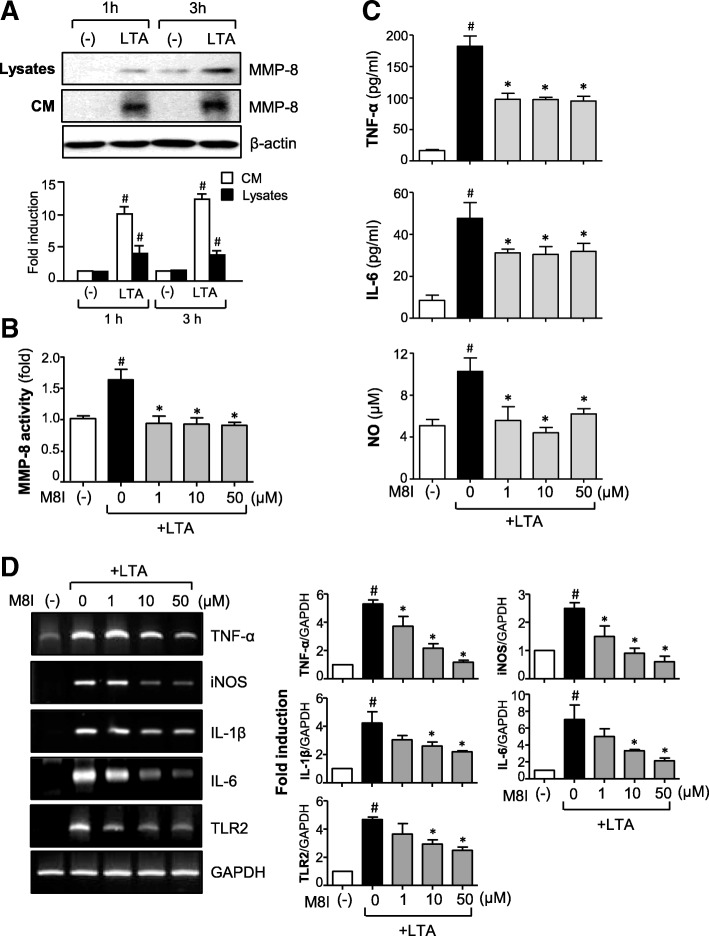
M8I suppressed the expression of pro-inflammatory molecules in LTA-stimulated astrocytes. **a** Cells were treated with LTA (10 μg/ml) for 1 or 3 h. MMP-8 protein expression was detected by Western blot in conditioned media (CM) and cell lysates. Representative blots (upper panel) and quantification data (lower panel) are shown. **b** MMP-8 activity assay data. Primary astrocytes were treated with M8I for 1 h before stimulation with LTA (10 μg/ml). One hour later, the supernatants were collected to measure MMP-8 activity using the SensoLyte® 520 MMP-8 assay system. **c** The effect of M8I on TNF-α, IL-6, and NO production in cells treated with LTA for 24 h. **d** The effect of M8I on the mRNA expression of iNOS, cytokines, and TLR2 was determined by RT-PCR analysis. The data are the mean ± S.E.M. of three independent experiments. ^#^*P* < 0.05, vs. control samples. *P < 0.05, vs. LTA-treated samples

Our previous studies demonstrated that MMP-8 modulates neuroinflammation in cultured microglia, septic mice, and cerebral ischemia [23, 24]. To determine whether MMP-8 also plays a role in LTA-mediated inflammatory responses in astrocytes, we examined the effect of M8I on the expression of various inflammatory molecules. First, we confirmed the protein expression of MMP-8 in LTA-stimulated astrocytes—both in cell lysates and conditioned media (Fig. 2a). LTA also increased the enzymatic activity of MMP-8, which was blocked by M8I (Fig. 2b). We found that treatment with M8I significantly inhibited the production of TNF-α, IL-6, and NO in LTA-stimulated astrocytes (Fig. 2c). Moreover, M8I suppressed the mRNA expression of TNF-α, iNOS, IL-1β, IL-6, and TLR2 induced by LTA (Fig. 2d). M8I did not have any cytotoxicity in the concentrations used for at least 48 h (data not shown). The results imply the role of MMP-8 as a proinflammatory mediator in activated astrocytes.

### M8I suppresses LTA-induced NF-κB activity with increasing PPAR-γ activity

**Fig. 3:**
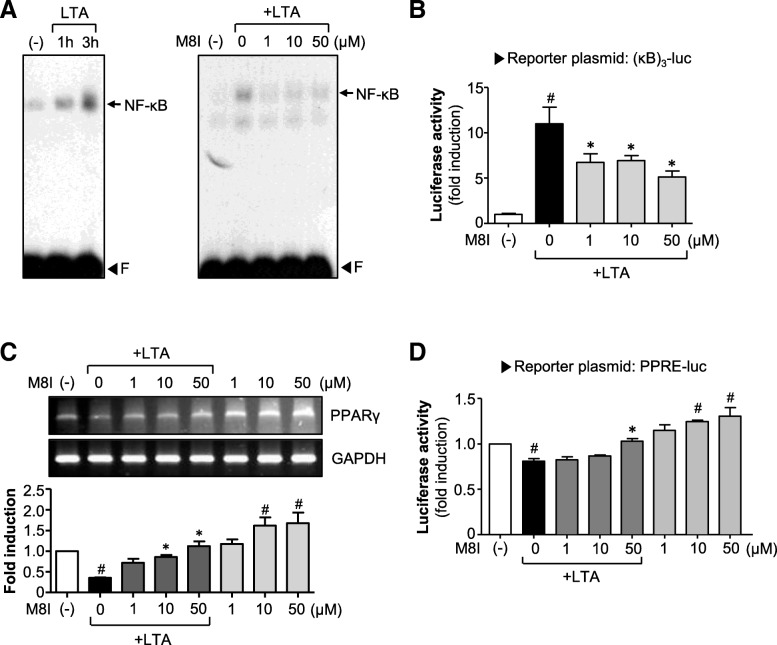
M8I suppressed the DNA binding and transcriptional activities of NF-κB, while it increased PPAR-γ expression and transcriptional activities in astrocytes. **a** EMSA data shows that NF-κB DNA binding activity was increased in astrocytes treated with LTA for 1 and 3 h (left panel). M8I significantly inhibited NF-κB DNA binding activity in cells treated with LTA for 3 h (right panel). The arrow indicates a DNA–protein complex of NF-κB. “F” indicates a free probe. **b** Effect of M8I on the reporter gene activity of NF-κB. **c** Effect of M8I on PPAR-γ expression. Primary astrocytes were pretreated with M8I for 1 h prior to incubation with LTA (10 μg/ml) for 6 h. The mRNA level of PPAR-γ was determined by RT-PCR. Quantification data are shown in the bottom panel. **d** Effect of M8I on PPRE-luc reporter gene activity. The data are the mean ± S.E.M. of three independent experiments. ^#^*P* < 0.05, vs. control samples. *P < 0.05, vs. LTA-treated samples

To further investigate the anti-inflammatory mechanism of M8I, we examined the effect of M8I on NF-κB, a key transcription factor modulating proinflammatory gene expression in various immune cells [29]. We found that LTA increased the DNA binding activity of NF-κB, which was inhibited by M8I (Fig. 3a). M8I also suppressed NF-κB-mediated transcription activity as shown by the reporter gene assay (Fig. 3b). Next, we examined the effect of M8I on PPAR-γ, a nuclear receptor that acts as an anti-inflammatory regulator in astrocytes and microglia [30, 31]. We found that the treatment of astrocytes with LTA significantly suppressed PPAR-γ expression. The treatment with M8I, however, restored PPAR-γ expression to near normal levels (Fig. 3c). Moreover, treatment with M8I without LTA also increased the expression of PPAR-γ. Next, to investigate whether M8I increases the transcriptional activity of PPAR-γ, a cell-based reporter gene assay was performed. We found that M8I increased PPRE-luc activity in both the presence and absence of LTA (Fig. 3d).

### M8I inhibits LTA-induced phosphorylation of MAP kinases and Akt

**Fig. 4:**
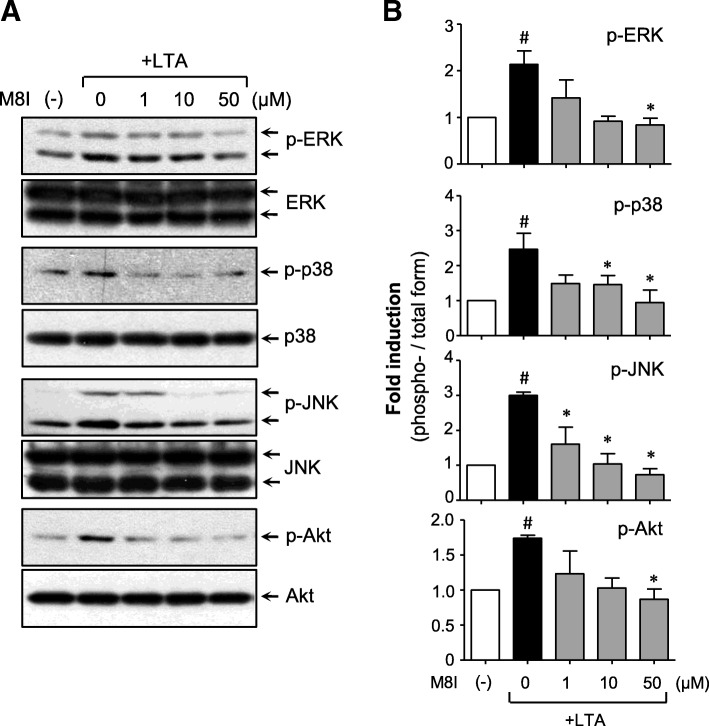
M8I inhibited the phosphorylation of MAP kinases and Akt in LTA-stimulated astrocytes. **a** Western blot analysis for MAP kinases and Akt activities. Cell extracts were prepared from astrocytes treated with LTA (10 μg/ml) for 1 h in the presence or absence of M8I and then subjected to immunoblot analysis using antibodies against the phospho- or total forms of three types of MAPKs or Akt. The autoradiograms are representative of three independent experiments. **b** Quantification of Western blot data. Levels of the phosphorylated forms of MAP kinases and Akt were normalized with respect to the level of each total form and expressed as relative fold changes versus the control group. The data are the means ± S.E.M. for three independent experiments. ^#^*P* < 0.05, vs. control samples. *P < 0.05, vs. LTA-treated samples

Next, we examined the effects of M8I on MAP kinases and Akt, which play an important role in proinflammatory gene expression by modulating transcription factors such as NF-κB [11]. We found that LTA induced the phosphorylation of three types of MAP kinase and Akt, and treatment with M8I markedly suppressed the phosphorylation of all these kinases in LTA-stimulated astrocytes (Fig. 4). These results suggest that MAP kinase and PI3K/Akt signaling pathways are involved in the anti-inflammatory action of M8I in LTA-stimulated astrocytes.

### M8I shows antioxidant effects by inhibiting ROS production and increasing phase II antioxidant enzyme expression via Nrf2/ARE signaling axis

**Fig. 5:**
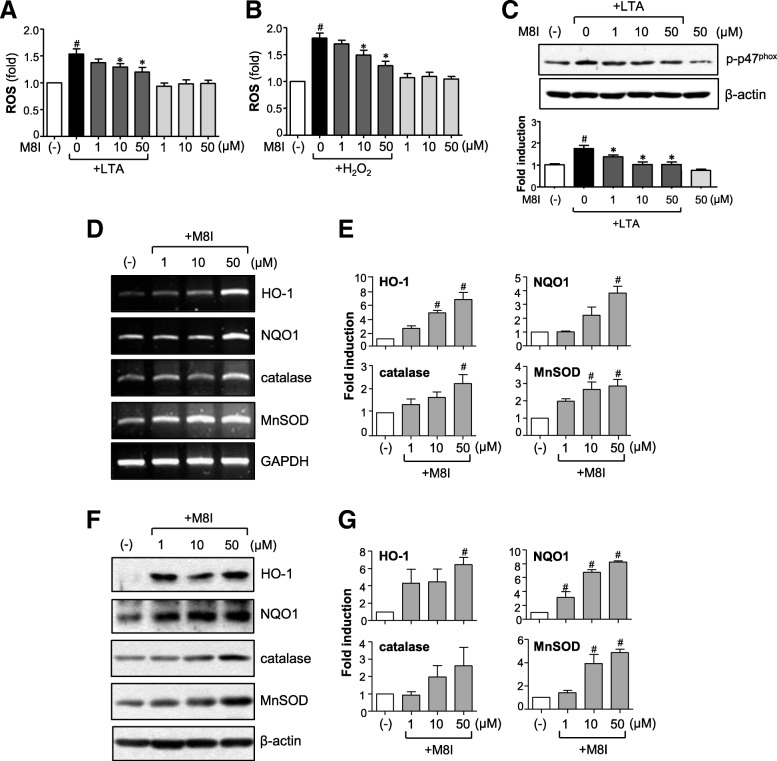
M8I inhibited ROS production and increased antioxidant enzyme expression. **a**, **b** Effect of M8I on ROS production in **a** LTA or **b** H_2_O_2_-stimulated astrocytes. Cells were pretreated with M8I for 1 h, followed by treatment with LTA (10 μg/ml) or H_2_O_2_ (500 μM) for 30 min. Intracellular ROS levels were measured by the DCF-DA method. **c** Western blot analysis for phosphorylation of the p47^phox^ subunit of NADPH oxidase. Cells were pre-treated with M8I for 1 h, followed by LTA (10 μg/ml, for 15 min), and then subjected to immunoblot analysis using antibodies against phospho-p47^phox^. Quantification data are shown in the graph (*n* = 3). **d** Effect of M8I on the mRNA expression of HO-1, NQO1, catalase, and MnSOD. **e** Quantification of RT-PCR data (*n* = 3). **f** Effect of M8I on the protein expression of HO-1, NQO1, catalase, and MnSOD. **g** Quantification of Western blot analysis (*n* = 3). ^#^*P* < 0.05, vs. control samples. *P < 0.05, vs. LTA-treated samples

**Fig. 6:**
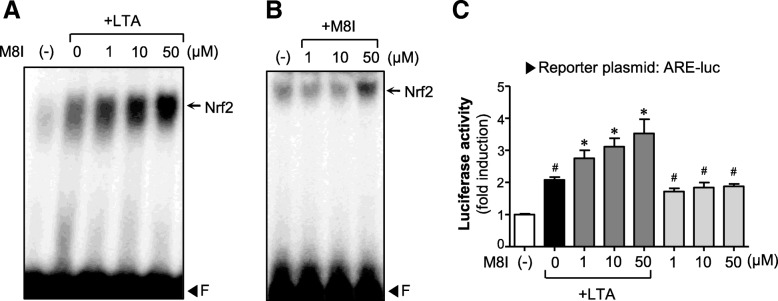
M8I increased the DNA binding and transcriptional activities of Nrf2. **a**, **b** EMSA for Nrf2 DNA binding activity. Nuclear extracts were prepared from astrocyte cells treated with M8I in the presence or absence of LTA (10 μg/ml) for 6 h and incubated with the probe (ARE). The arrow indicates a DNA–protein complex of Nrf2. **c** Effect of M8I on ARE-luc reporter gene activity. Astrocytes were transfected with the reporter plasmid (ARE-luc) and treated with M8I in the presence or absence of LTA for 6 h, and luciferase assay was performed. The data are the means ± S.E.M. for three independent experiments. ^#^*P* < 0.05, vs. control samples. ^*^P < 0.05, vs. LTA-treated samples

ROS are the byproducts of respiration and, at low levels, essential for biological function. However, excessive ROS give rise to inflammation-associated neurological disorders, including AD and PD [10, 32]. In the present study, we found that M8I inhibited intracellular ROS levels in LTA or H_2_O_2_-treated astrocytes, suggesting the antioxidant role of M8I in astrocytes (Fig. 5a, b). M8I also inhibited the phosphorylation of p47^phox^ (Fig. 5c), a key component of NADPH oxidase complex responsible for ROS release in astrocytes [10]. To further investigate the antioxidant mechanism of M8I, we examined the effects of M8I on phase II antioxidant enzymes that are under the control of Nrf2/ARE signaling. We observed that M8I increased the expression of HO-1, NQO1, catalase, and MnSOD at both mRNA and protein levels (Fig. 5d–g). Next, we examined the effects of M8I on Nrf2/ARE signaling. EMSA data showed that M8I increased Nrf2 DNA binding activity in the absence or presence of LTA (Fig. 6a, b). The reporter gene assay employing ARE-luc also showed that M8I enhanced Nrf2-mediated transcriptional activities (Fig. 6c). The data suggest that upregulation of Nrf2/ARE-mediated antioxidant enzyme expression may contribute to antioxidant effects of M8I in astrocytes.

### M8I increases the expression and nuclear translocation of Nrf2 and c-Jun in astrocytes

**Fig. 7:**
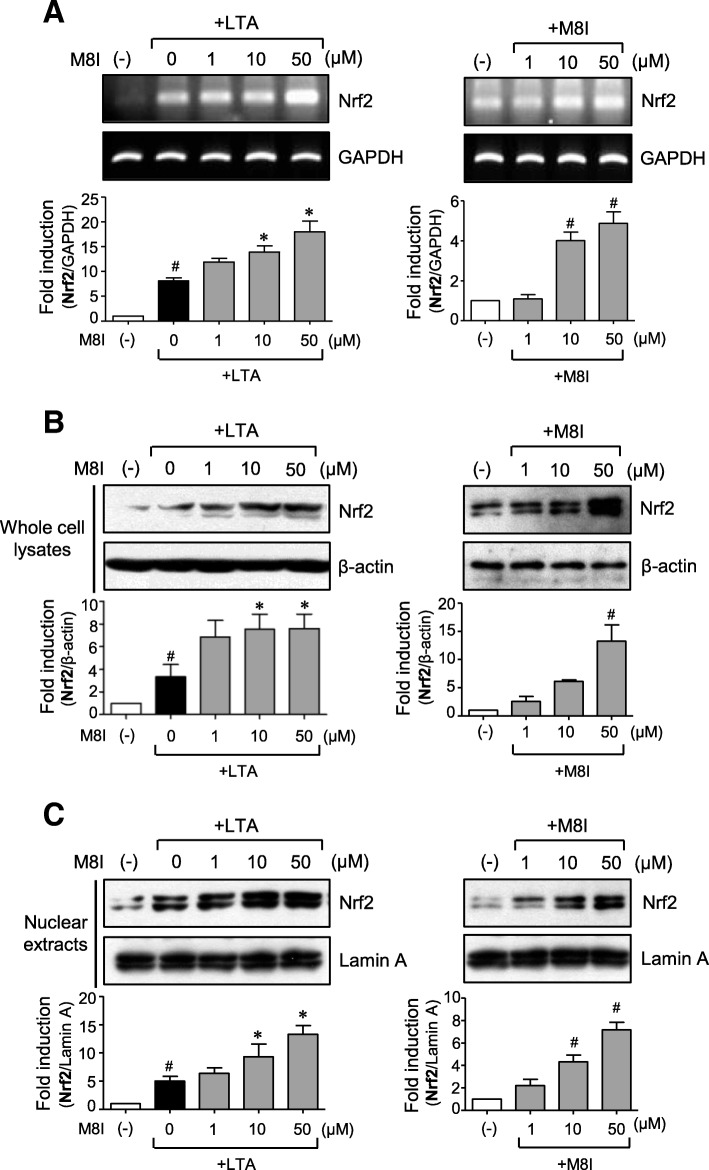
M8I increased the expression and nuclear translocation of Nrf2 in astrocytes. Cells were treated with M8I in the presence or absence of LTA for 3 h. **a** The effect of M8I on Nrf2 mRNA expression was determined by RT-PCR analysis. **b**, **c** The protein levels of Nrf2 in **b** whole cell lysates and **c** nuclear extracts were determined by Western blot analysis. Quantification data are shown in the bottom panels. The data are the means ± S.E.M. for three independent experiments. ^#^*P* < 0.05, vs. control samples. *P < 0.05, vs. LTA-treated samples

**Fig. 8:**
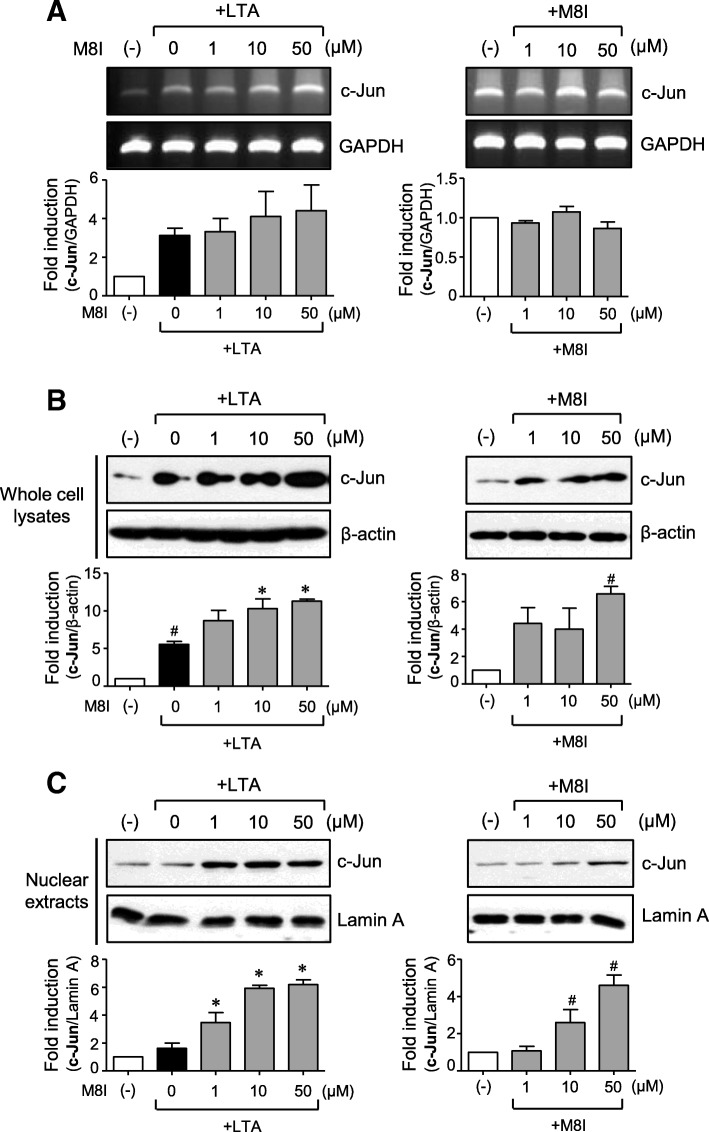
M8I increased the expression and nuclear translocation of c-Jun in astrocytes. Cells were treated with M8I in the presence or absence of LTA for 3 h. **a** The effect of M8I on c-Jun mRNA expression was determined by RT-PCR analysis. **b**, **c** The protein levels of c-Jun in **b** whole cell lysates and **c** nuclear extracts were determined by Western blot analysis. Quantification data are shown in the bottom panels. The data are the means ± S.E.M. for three independent experiments. ^#^*P* < 0.05, vs. control samples. *P < 0.05, vs. LTA-treated samples

We previously reported that Nrf2 and c-Jun coordinately regulate the expression of phase II antioxidant enzyme genes in rat primary astrocytes [33]. Therefore, in the present study, we examined the effect of M8I on Nrf2 and c-Jun expression. RT-PCR and Western blot analysis showed that M8I increased the expression of Nrf2 at both the mRNA and protein levels (Fig. 7a, b). Interestingly, M8I increased Nrf2 expression both in the absence and presence of LTA. Subsequently, nuclear translocation of Nrf2 was increased in M8I-treated cells (Fig. 7c). Unlike Nrf2, the mRNA expression of c-Jun was not significantly altered by M8I (Fig. 8a). However, M8I increased the protein expression and nuclear translocation of c-Jun (Fig. 8b, c). These data suggest that M8I modulates Nrf2 expression at the transcriptional level and c-Jun at the post-transcriptional level. Therefore, concomitant upregulation of Nrf2 and c-Jun by M8I may contribute to enhanced antioxidant enzyme expression in astrocytes.

## Discussion

The present study demonstrated the anti-inflammatory and antioxidant effects of MMP-8 inhibitor in LTA-stimulated rat primary astrocytes. M8I inhibited various inflammatory molecules such as iNOS and cytokines such as TNF-α, IL-1β, and IL-6. Interestingly, M8I suppressed the expression of TLR2, the cell surface receptor of LTA, which may lead to attenuation of TLR2-mediated inflammatory signaling. Through mechanistic studies, we demonstrated that M8I exerts anti-inflammatory effects by blocking the MAPK, PI3K/Akt, and NF-κB and enhancing of PPAR-γ activities. In addition, M8I showed antioxidant effects by upregulating phase II antioxidant enzyme expression via the Nrf2/ARE signaling pathway. In particular, M8I upregulated the expression of Nrf2 and c-Jun, which coordinately regulate the expression of antioxidant genes. The data collectively suggest the therapeutic potential of M8I for neuroinflammatory disorders that are associated with reactive astrocytes as well as microglial activation.

Recent studies reported that the astrocyte reactivity is regulated by multiple signaling pathways including JAK/STAT3, NF-κB, CN/NFAT, and MAP kinases [3, 4]. NF-κB activation in astrocytes plays an important role in chronic inflammation and the progression of neurodegenerative diseases. The JAK/STAT3 pathway appears to mediate the action of the A2 reactive astrocytes. However, the role of the JAK/STAT3 pathway in reactive astrocytes is rather controversial. Thus, modulation of these pathways has been suggested to be a potential therapeutic strategy for neurodegenerative diseases [3, 4]. In the present study, we found that M8I inhibited DNA binding and transcriptional activities of NF-κB in LTA-stimulated astrocytes. In addition, M8I inhibited three types of MAP kinases, which are also involved in astrocyte reactivity. Thus, the inhibition of NF-κB and MAP kinases appears to result in downregulation of inflammatory molecules such as iNOS and cytokines.

In this study, we found that M8I increased the expression of phase II antioxidant enzymes such as HO-1, NQO1, catalase, and MnSOD, which are governed by Nrf2/ARE signaling [34, 35]. The ARE-regulated genes are preferentially activated in astrocytes, which consequently have more efficient detoxification and antioxidant defenses than neurons [36]. Previous studies have reported that astrocyte-specific overexpression of Nrf2 protects against neurodegeneration in mouse models of AD, PD, ALS, HD, and MS, suggesting that Nrf2 is a crucial therapeutic target for the treatment of many neurodegenerative diseases [37–40]. The results in the present study showed that M8I dramatically increased the expression and nuclear translocation of Nrf2 along with its counterpart c-Jun and subsequently increased the expression of antioxidant enzymes in astrocytes. Further studies are necessary to determine whether M8I-mediated upregulation of Nrf2 in astrocytes endows neuroprotection in diseased animal models.

The anti-inflammatory role of Nrf2 also has been reported by many papers [41]. In particular, HO-1 is a well-known anti-inflammatory and antioxidant enzyme that is regulated through the Nrf2/ARE signaling pathway [42]. We have previously reported that the upregulation of HO-1 plays a key role in mediating the anti-inflammatory and antioxidant mechanism in LPS-stimulated microglia [43]. Carbon monoxide, one of the products of HO-1, has been reported to inhibit TLR signaling through suppression of NADPH oxidase-dependent ROS generation [44]. Therefore, the potentiation of Nrf2 and HO-1 by M8I may contribute to the anti-inflammatory and antioxidant effects of M8I in LTA-stimulated astrocytes.

Previous studies have reported that PPAR-γ regulates the inflammatory responses mediated by astrocytes and microglia, protects neurons from damage, reduces oxidative stress, and improves mitochondrial function [30, 45]. PPAR-γ has been reported to inhibit NF-κB activation and suppress the expression of proinflammatory molecules [31, 46]. In addition, the interaction between PPAR-γ and Nrf2 has been reported by many papers [47, 48]. Moreover, the effect of Nrf2 in ameliorating oxidative stress has been proposed to inhibit NF-κB [49]. Due to the interactions among PPAR-γ, Nrf2, and NF-κB, it has been proposed that coactivation of Nrf2 and PPAR-γ may improve the therapeutic outcome of neurological disorders [31]. In the present study, M8I increased PPAR-γ and Nrf2 with a decrement of NF-κB activities in astrocytes. Based on the previous studies, we speculate that PPAR-γ may be involved in M8I-mediated anti-inflammatory and antioxidant effects in astrocytes by modulating NF-κB and Nrf2 signaling pathways.

In our previous study on the anti-inflammatory effect of an MMP-8 inhibitor in activated microglia, we reported that M8I specifically inhibited TNF-α processing and protease-activated receptor-1 activation, leading to blockage of inflammatory signaling [23, 50]. Moreover, M8I inhibited microglial activation and TNF-α expression in the brain of mice with systemic inflammation and cerebral ischemia, corroborating the findings through MMP-8 knockdown experiments [23, 24]. Although most of the data from experiments involving microglia and astrocytes indicated that the effects of M8I were largely mediated through MMP-8 inhibition, we cannot rule out the possibility of involvement of some nonspecific effects of M8I. Studies to clarify the MMP-8-dependent and MMP-8-independent effects of M8I in reactive astrocytes as well as microglia are necessary in future.

## Conclusions

The present study reports, for the first time, the anti-inflammatory and antioxidant effects of an MMP-8 inhibitor in LTA-stimulated astrocytes. We demonstrated that M8I decreased proinflammatory molecules with an increment of antioxidant enzymes, in which NF-κB/Nrf2/PPAR-γ signaling pathways are involved. Since reactive astrocytes play a crucial role in neuroinflammation and neuronal cell death, controlling astrocyte reactivity by M8I may provide therapeutic potential for neurodegenerative diseases.

## Additional file

Additional file 1: **Figure S1**. Quantitative real-time PCR (RT-qPCR) data showing the expressions of iNOS, COX-2, cytokines, TLR2, and MMPs in LTA-simulated astrocytes. (PDF 438 kb)

## Abbreviations

ARE: Antioxidant response element
EMSA: Electrophoretic mobility shift assay
ERK: Extracellular signal-regulated kinase
HO-1: Heme oxygenase-1
iNOS: Inducible nitric oxide synthase
JNK: c-Jun N-terminal kinase
LTA: Lipoteichoic acid
M8I: MMP-8 inhibitor
MAPK: Mitogen-activated protein kinase
MMP: Matrix metalloproteinase
MnSOD: Manganese-dependent superoxide dismutase
NF-κB: Nuclear factor-κB
NQO1: NAD(P)H:quinone oxidoreductase 1
Nrf: Nuclear factor-E2-related factor
PPAR-γ: Peroxisome proliferator-activated receptor-γ
ROS: Reactive oxygen species
