# Supplementary material for: Anti-inflammatory and anti-oxidant mechanisms of an MMP-8 inhibitor in lipoteichoic acid-stimulated rat primary astrocytes: involvement of NF-κB, Nrf2, and PPAR-γ signaling pathways
Source: J Neuroinflammation. 2018 Nov 23;15:326. doi: 10.1186/s12974-018-1363-6 (PMC6260848; doi:10.1186/s12974-018-1363-6)
Supplement: Supplementary file 1 — Figure S1. Quantitative real-time PCR (RT-qPCR) data showing the expressions of iNOS, COX-2, cytokines, TLR2, and MMPs in LTA-simulated astrocytes. (PDF 438 kb) [file 12974_2018_1363_MOESM1_ESM.pdf]

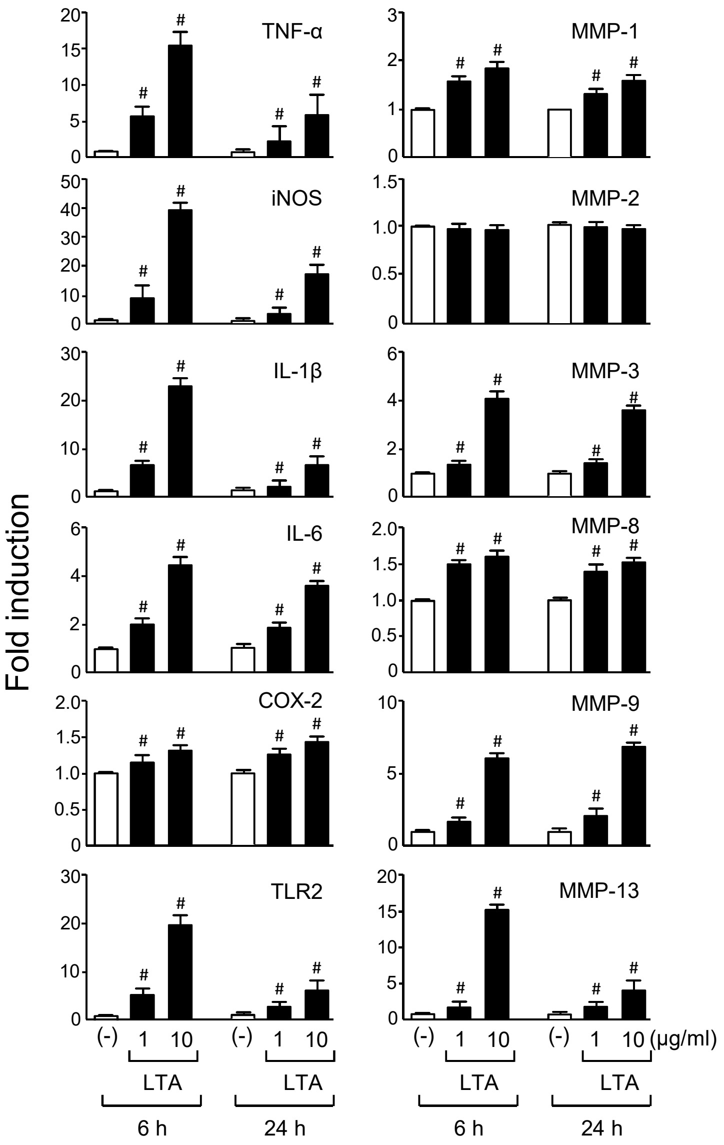

Figure S1: **Quantitative real time PCR (RT-qPCR) data showing the expressions of iNOS, COX-2, cytokines, TLR2, and MMPs in LTA-simulated astrocytes.** The data are the mean ± S.E.M. of three independent experiments. #*P* < 0.05, vs. control samples.
